# GOBP1 from the Variegated Cutworm *Peridroma saucia* (Hübner) (Lepidoptera: Noctuidae) Displays High Binding Affinities to the Behavioral Attractant (*Z*)-3-Hexenyl acetate

**DOI:** 10.3390/insects12100939

**Published:** 2021-10-15

**Authors:** Ya-Lan Sun, Jun-Feng Dong, Yue-Qin Song, Shao-Li Wang

**Affiliations:** 1 College of Horticulture and Plant Protection, Henan University of Science and Technology, Luoyang 471000, China; yalansun@haust.edu.cn (Y.-L.S.); songyueqin6@haust.edu.cn (Y.-Q.S.); 2 Department of Plant Protection, Institute of Vegetables and Flowers, Chinese Academy of Agricultural Sciences, Beijing 100081, China

**Keywords:** *Peridroma saucia*, general odorant-binding protein, real-time quantitative PCR, Western blot, fluorescence competitive binding, EAG recording, behavioral response

## Abstract

**Simple Summary:**

The variegated cutworm *Peridroma saucia* (Hübner) is a polyphagous pest that originated in Europe and North America and has gradually become an important agricultural pest worldwide since the 1970s. In 2017, this pest experienced a serious outbreak in the suburbs of Luoyang, Henan Province, China. Odorant-binding proteins (OBPs) are small soluble proteins expressed in insect olfactory tissues and involved in the first step of odorant reception. Characterization of the function of *P*. *saucia* OBPs may contribute to identifying new attractants/repellents and assist in the development of more efficient and environmentally acceptable controlling strategies of this pest. In this study, we cloned an antennae-specific gene *PsauGOBP1* which encodes for a general odorant-binding protein (GOBP) of *P*. *saucia*. We expressed PsauGOBP1 in bacteria and found that the purified recombinant PsauGOBP1 binds robustly to (*Z*)-3-hexenyl acetate. Electroantennogram tests demonstrate that (*Z*)-3-hexenyl acetate elicits strong responses from the antennae of both males and females of *P*. *saucia*. Y-tube olfactometer bioassays show that (*Z*)-3-hexenyl acetate is attractive to adult *P*. *saucia* of both sexes. This study increases our understanding of the olfactory mechanism of *P*. *saucia* and provides a theoretical basis for the design of attractants for the control of *P*. *saucia*.

**Abstract:**

The variegated cutworm *Peridroma saucia* (Hübner) is a worldwide pest that causes serious damage to many crops. To recognize sex pheromones and host plant volatiles, insects depend on olfactory chemoreception involving general odorant-binding proteins (GOBPs). In this study, *PsauGOBP1* was cloned from the adult antennae of *P. saucia*. RT-qPCR and Western-blot analysis showed that PsauGOBP1 was specifically and equally expressed in the adult antennae of both females and males. Fluorescence competitive-binding assays with sex pheromones and host plant volatiles demonstrated that PsauGOBP1 bound to six host plant volatiles: (*Z*)-3-hexenyl acetate (K_D_ = 4.0 ± 0.1 μM), citral (K_D_ = 5.6 ± 0.4 μM), farnesol (K_D_ = 6.4 ± 0.6 μM), nonanal (K_D_ = 6.8 ± 0.3 μM), (*Z*)-3-hexen-1-ol (K_D_ = 8.5 ± 0.6 μM), and benzaldehyde (K_D_ = 9.4 ± 0.5 μM). Electroantennogram recordings with the six host plant volatiles indicated that (*Z*)-3-hexenyl acetate elicited the strongest responses from both male and female antennae. Further bioassays using Y-tube olfactometers showed that (*Z*)-3-hexenyl acetate was attractive to adult *P. saucia* of both sexes. These results suggest that PsauGOBP1 might be involved in detecting host plant volatiles and that (*Z*)-3-hexenyl acetate might serve as a potential attractant for the biological control of *P. saucia*.

## 1. Introduction

Insects depend on olfactory chemoreception for locating reproductive partners, food sources, and oviposition sites, and also for avoiding predators [1]. As a consequence, insects have evolved a highly sensitive and sophisticated olfactory system in order to deal with their ever-changing chemical environment [2,3,4]. In peri-receptor events, odorant molecules pass through the aqueous sensillum lymph before reaching the dendrites of olfactory receptor neurons. As they are hydrophobic molecules with low solubility in the sensillum lymph, odorants are bound and transported by a group of soluble carrier proteins termed odorant-binding proteins (OBPs) [5,6,7,8,9].

The first insect OBP was discovered in the giant moth *Antheraea polyphemus* by Vogt and Riddiford [10]. The latter authors found that a small soluble protein, which was abundant in the sensillum lymph of *A. polyphemus* antennae, bound to radioactive sex pheromones; the protein was therefore named pheromone-binding protein (PBP). With the development of gene cloning and transcriptome/genome sequencing in the following 40 years, more than 400 OBPs have been identified from more than 40 insect species [11,12], such as *Bombyx mori* [13,14], *Drosophila melanogaster* [15,16], *Anopheles gambiae* [17,18], *Apis mellifera* [19], *Helicoverpa armigera* [20,21,22], and *Tribolium castaneum* [23]. The most typical feature of OBP sequences are the six highly conserved cysteines that form three disulfide bridges to ensure a compact three-dimensional structure [24,25]. However, OBPs with fewer or more conserved cysteines have also been found [26,27,28,29]. OBPs can be divided into three distinct subfamilies: minus-C OBPs with four conserved cysteine residues; classic OBPs with six conserved cysteines, such as PBPs and general-odorant binding proteins (GOBPs); plus-C OBPs with eight conserved cysteines.

Among moth species, PBPs and GOBPs are numerically dominant. PBPs are usually detected in the pheromone-sensitive sensilla trichodea and mainly bind sex pheromones that are a blend of compounds emitted by female sex pheromone glands to mediate (attract/repel) male behavior [30,31,32]. GOBPs, which are further classified into GOBP1 and GOBP2 [33,34], are usually located in general odorant-sensitive sensilla basiconica and are thought to detect general odorants such as volatiles from host plants and oviposition sites [35,36]. For instance, Northern blot analysis of GOBPs in *Manduca sexta*, *A*. *polyphemus*, *B*. *mori*, and *A. pernyi* showed that the GOBPs were associated with general odorant-sensitive sensilla basiconica [37]. Later studies using in situ hybridization and immunolocalization demonstrated that moth GOBPs are also expressed in the pheromone-sensitive sensilla trichodea [38,39]. A study of *Agrotis ipsilon*, for example, revealed that AipsGOBP1 and AipsGOBP2 were expressed and co-localized in both sensilla basiconica and sensilla trichodea [40]. A recent study of GOBPs in *H*. *armigera* using immunofluorescent staining, however, showed that HarmGOBP1 and HarmGOBP2 were restricted to sensilla basiconica [41]. In addition, competitive fluorescence binding assays have also suggested that GOBPs are functionally divergent. GOBPs displayed strong binding affinities with their host plant volatiles for some insect species [42,43] but with sex pheromones for other insect species [44,45]. Moreover, a few studies reported that some GOBPs showed high binding affinities for both host plant volatiles and sex pheromone components [46,47] or even insecticides [48]. Therefore, the functional specificity of GOBPs remains unclear. Functional characterization of GOBPs from additional insect species is needed.

*Peridroma saucia* Hübner (Lepidoptera, Noctuidae), also known as the variegated cutworm, is a highly polyphagous pest that can feed on more than 121 plant species including maize, cotton, tobacco, and soybean [49]. This pest was first recorded in Europe in 1790 and remains a major pest in North America and Europe [50,51,52]. Since the 1970s, *P. saucia* had been spreading as an invasive species in Korea and Japan and has gradually become an important agricultural pest worldwide [53,54,55]. In China, the first outbreak of *P*. *saucia* occurred in Sichuan Province in 1981 [56]. It has since been found in more than 12 provinces in China [57,58,59]. In the suburbs of Luoyang (Luanchuan County, Henan Province, China) in 2017, *P. saucia* damaged more than 6000 ha of agricultural crops and reduced yields by more than 50% in the most severely affected soybean fields [60]. One potential way to manage this pest is via olfaction-based control [61]. Behavioral analysis of insect responses to volatile organic compounds by plants may contribute to identifying new attractants/repellents [62]. At present, however, studies on *P*. *saucia* chemoreception are limited to antennal transcriptome analyses and chemosensory gene identification [60]. The development of olfaction-based control techniques will require the characterization of the function of *P*. *saucia* GOBPs and other olfactory proteins.

In this study, we cloned the full-length gene of *P*. *saucia*, *GOBP1* (*PsauGOBP1*), and expressed it in *Escherichia coli*. We then used real-time quantitative PCR (RT-qPCR) and Western blot analysis to assess the expression patterns of PsauGOBP1 in different tissues of the insect. We subsequently measured the ligand-binding activities of PsauGOBP1 with sex pheromones and host plant volatiles using a fluorescence competitive-binding assay. Finally, we used electroantennogram recordings and behavior analyses to determine whether the odorant molecules that exhibited high binding affinities elicit physiological and behavioral responses in *P*. *saucia*. The results obtained increase our understanding of the function of lepidopteran GOBPs and should be useful for developing olfaction-based control strategies of *P*. *saucia.*

## 2. Materials and Methods

### 2.1. Insect Rearing and Tissue Collection

Adult *P*. *saucia* were collected from Luoyang, Henan Province, China. Forty adults in a sex ratio of 1:1 were kept in a cage (25 cm in diameter, 40 cm in length) for mating and oviposition. The larvae that hatched from the eggs were kept in a rearing room (27 ± 1 °C, with 70% ± 5% relative humidity and a 16 h L/8 h D cycle) and were fed an artificial diet [52]. Male and female pupae were placed in cages separately for eclosion. Adults were given a 10% (*v*/*v*) honey solution.

For gene cloning and RT-qPCR, antennae, proboscises, tarsi, wings, pheromone glands, and hair brushes were collected separately from both sexes of 3-day-old adult *P*. *saucia*. Collected samples were immediately placed in 1.5 mL Eppendorf tubes, frozen in liquid nitrogen, and then stored at −70 °C.

### 2.2. RNA Extraction and cDNA Synthesis

Total RNA from different tissues of *P*. *saucia* was extracted with the RNeasy Plus Mini Kit and following the manufacturer’s instructions (Qiagen, Venlo, the Netherlands). The quantity of RNA samples was determined by 1.5% agarose gel electrophoresis and with a Nano Drop 2000 spectrophotometer (Nano-Drop Products, Wilmington, DE, USA). Total RNA from different tissues was first treated with DNase I (Takara, Dalian, China) to remove residual genomic DNA, and cDNA was then generated using M-MLV Reverse Transcriptase (Promega, Madison, VA, USA) and oligo-dT primer (Takara). The newly synthesized cDNA was used as a template for gene cloning and RT-qPCR analyses.

### 2.3. PsauGOBP1 Cloning and Sequencing

Full-length *PsauGOBP1* was amplified by PCR with ExTaq DNA polymerase (TaKaRa) under the following reaction conditions: 94 °C for 3 min; 30 cycles of 94 °C for 30 s, 55 °C for 30 s, and 72 °C for 30 s; followed by 72 °C for 2 min. The gene-specific primers used for PCR are listed in Table S1. The PCR products were first ligated into the pGEM-T easy vector (Promega). After transformation of *E*. *coli* Top10 competent cells with the ligation products, positive colonies were selected by PCR using the primers SP6 and T7; the colonies were grown in LB/ampicillin medium and were custom sequenced at Sangon Biotech, Shanghai, China.

### 2.4. Sequence Analysis and Phylogenetic Tree Construction

The signal peptide of PsauGOBP1 was predicted with SignalP-5.0 Server (http://www.cbs.dtu.dk/services/SignalP/, accessed in 1 July 2019). Sequence alignments were produced using Clustal Omega (https://www.ebi.ac.uk/Tools/msa/clustalo/, accessed in 1 July 2019). For phylogenetic tree construction, amino acid sequences of GOBPs and PBPs from different lepidopteran species were first aligned with ClustalX software [63] before an un-rooted neighbor-joining tree was constructed using MEGA7.0 [64] and visualized with Figtree (v1.4.3). The evolutionary distances were computed with the Jones–Taylor–Thornton (JTT) matrix-based method [65]. Bootstrap support of tree branches was assessed by resampling amino acid positions 1000 times. Accession numbers of GOBP and PBP sequences used in the tree construction are listed in Table S2.

### 2.5. RT-qPCR

RT-qPCR was used to evaluate the expression levels of *PsauGOBP1* in different tissues of *P*. *saucia*. Operations were carried out following the manufacturer’s instructions for SYBR Premix ExTaq II (Tli RNaseH Plus, Takara, Dalian, China) using the StepOne Plus Real-time PCR System (Applied Biosystems, Foster City, CA, USA). The reaction conditions were as follows: one cycle of 94 °C for 3 min; 38 cycles of 94 °C for 10 s and 60 °C for 30 s; followed by 94 °C for 1 min and 60 °C for 1 min. The *P*. *saucia* actin gene was chosen as the endogenous control and was used for normalizing target gene expression. Expression levels of *PsauGOBP1* were calculated using the 2^−ΔCt^ method [66]. Each reaction was performed in triplicate for each of three biological replicates. Tukey’s multiple comparison test after a one-way analysis of variance (ANOVA) was used to determine statistical differences for expression levels of *PsauGOBP1* in different tissues of *P*. *saucia*. All primers used in the experiment are listed in Table S1.

### 2.6. Expression and Purification of Recombinant PsauGOBP1

For the expression of recombinant PsauGOBP1, its coding region was amplified by PCR with specific primers preceded by NdeI or EcoRI restriction site. The PCR product was first cloned into the pGEM-T easy vector (Promega). The pGEM-T plasmid containing the sequence encoding the mature protein was then digested with NdeI and EcoRI restriction enzymes for 2 h at 37 °C. The digestion product was purified from the agarose gel using the TaKaRa MiniBEST Agarose Gel DNA Extraction Kit and was then ligated into the expression vector pET30b (Novagen, Darmstadt, Germany). BL21 *E*. *coli* cells were then transformed with the ligation products. Protein expression was induced by addition of IPTG to a final concentration of 0.4 mM when the optical density of the culture (OD_600_) had reached 0.8. Cells were grown for an additional 2–4 h at 37 °C, and then were harvested by centrifugation and sonication. After centrifugation, PsauGOBP1 was present as inclusion bodies. To solubilize the protein, the pellet from 1 L of culture was dissolved in 10 mL of 8 M urea containing 1 mM DTT in 50 mM Tris buffer (pH 7.4) and was then diluted to 100 mL with Tris buffer and dialyzed three times against Tris buffer. The protein was purified on QFF columns following standard protocols previously adopted for other odorant-binding proteins [67].

### 2.7. Native Protein Extraction and Western Blot Analysis

Rabbit antisera against PsauGOBP1 was prepared following our published protocol [68]. Antennae of both sexes were collected on the 3rd day after eclosion. The samples were first fully ground with 0.1% TFA (trifluoroacetic acid) and then centrifuged at 4 °C and 12,000 rpm for 20 min. The supernatant was collected for Western blot analysis. After electrophoretic separation under denaturing conditions (14% SDS-PAGE) of the protein extract, duplicate gels were stained with 0.1% Coomassie blue R250 in 10% acetic acid and 20% ethanol or were electroblotted on Trans-Blot nitrocellulose membranes (Bio-Rad Lab) following the procedure of Kyhse-Andersen [69]. The membrane was immersed in 2% skimmed milk overnight and was then incubated with the crude antiserum against the protein at a dilution of 1:500 (2 h). The membrane was then incubated with goat anti-(rabbit IgG) horseradish peroxidase conjugate (dilution 1:1000; 1 h). Immunoreacting bands were detected with 4-chloro-1-naphthol and hydrogen peroxide.

### 2.8. Fluorescence Competitive Binding Assay

Emission fluorescence spectra were recorded on a Hitachi F-4500 at 25 °C, with a 1 cm light path quartz cuvette and 5 nm slits for both excitation and emission. The protein was dissolved in 50 mM Tris-HCl buffer, pH 7.4, and ligands were added as 1 mM methanol solutions. To measure the affinity of the fluorescent ligand N-phenyl-1-naphthylamine (1-NPN) to PsauGOBP1, a 2 mM solution of the protein in 50 mM Tris-HCl, pH 7.4, was titrated with aliquots of 1 mM ligand in methanol to a final concentration of 16 µM. The probe was excited at 337 nm, and emission spectra were recorded between 380 and 450 nm. To analyze the binding affinity of other ligands to PsauGOBP1, a panel of 34 compounds including moth sex pheromones and host plant volatiles were used in the competitive-binding assay. The CAS number, purity, and company source of these compounds are listed in Table S3. A solution of the protein and 1-NPN, both at the concentration of 2 mM, was titrated with 1 mM methanol solutions of each competitor at a concentration of 12 (sex pheromones) or 16 µM (host plant volatiles).

The dissociation constant for 1-NPN and the stoichiometry of binding were obtained by processing the data with GraphPad Prism 6. Dissociation constants of the competitors were calculated from the corresponding IC_50_ values (concentrations of ligands that reduced the initial fluorescence value of 1-NPN by 50%), using the following equation: K_D_ = [IC_50_]/1 + [1-NPN]/K_1-NPN_, where [1-NPN] is the free concentration of 1-NPN, and K_1-NPN_ is the dissociation constant of the protein/1-NPN complex.

### 2.9. Electroantennogram (EAG) Recording

Individual synthetic nonanal, citral, farnesol, benzaldehyde, (*Z*)-3-hexenyl acetate, and (*Z*)-3-hexen-1-ol was diluted with paraffin oil to make each solution of 10 μg/μL. Each solution was added onto a filter paper (0.2 × 1 cm), and the final loading capacity doses of each solution were 100 μg. A 10 μL volume of paraffin oil was used as the control. Two- to three-day-old male and female *P*. *saucia* were used to obtain EAG recordings for the six volatiles. As described by Sun et al. with modification [70], the antennae were cut from the head and used for electro-signal recordings. After a few segments of the tip were cut off, the antenna was glued to the antenna holder (using conductive gel, Spectra 360, Parker Lab, NJ, USA), and the antenna holder was inserted into the EAG probe. The EAG signal was first amplified with a DC/AC preamplifier (Syntech UN-06), and was further processed with Autospike software (Syntech, Hilversum, the Netherlands).

The EAG data were analyzed using GraphPad Prism 6. The level of significance was set at *p* < 0.05. Unpaired Student’s *t*-tests were used to compare the EAG responses of male vs. female *P*. *saucia*. For comparison of responses to the six volatiles, the EAG data were first subjected to a one-way ANOVA; this was done separately for males and females. If the ANOVA was significant, means were compared with Tukey’s multiple comparison test.

### 2.10. Behavioral Responses of Peridroma saucia to (Z)-3-Hexenyl acetate

A Y-tube olfactometer was used to assess the behavioral responses of male and female *P. saucia* to (*Z*)-3-hexenyl acetate as described by Yan and Wang with modifications [71]. In brief, the system consisted of a central tube (20 cm long, 6 cm diameter) and two lateral arms (35 cm long, 6 cm diameter). (*Z*)-3-hexenyl acetate was dissolved in paraffin oil to obtain 10 µg/µL solutions. Filter paper (1 × 2 cm) with 10 µL of sample or 10 µL of paraffin oil was placed in a 250 mL flask. During the test, the room temperature was kept at 24 ± 2 °C with 70% ± 5% humidity and 0.6 lux of red light. Male or female moths were individually released at the base of the central tube and were observed for 5 min. If the moth did not make a choice within this period, it was removed and recorded as “no choice”. Moths that moved more than half way along one of the lateral arms and remained for at least 5 s were recorded as having made a choice for the odor offered through that arm. After five individuals were tested, the olfactometer was turned and the flasks were switched in order to avoid positional effects. Tested compounds and paraffin oil were renewed every 5–10 min. The olfactometer and the flask were cleaned with water, ethanol, and acetone at the end of each day.

*Chi*-squared analyses were performed to determine whether the numbers of *P*. *saucia* that made a choice differed between the two odor sources (a 50:50 probability was set for the percentage of moths selecting the control and the test sides. The moths that made no choice were not included in the statistical analysis).

## 3. Results

### 3.1. Sequence Analysis of PsauGOBP1 and Alignment to Orthologs from Other Species

**Figure 1:**
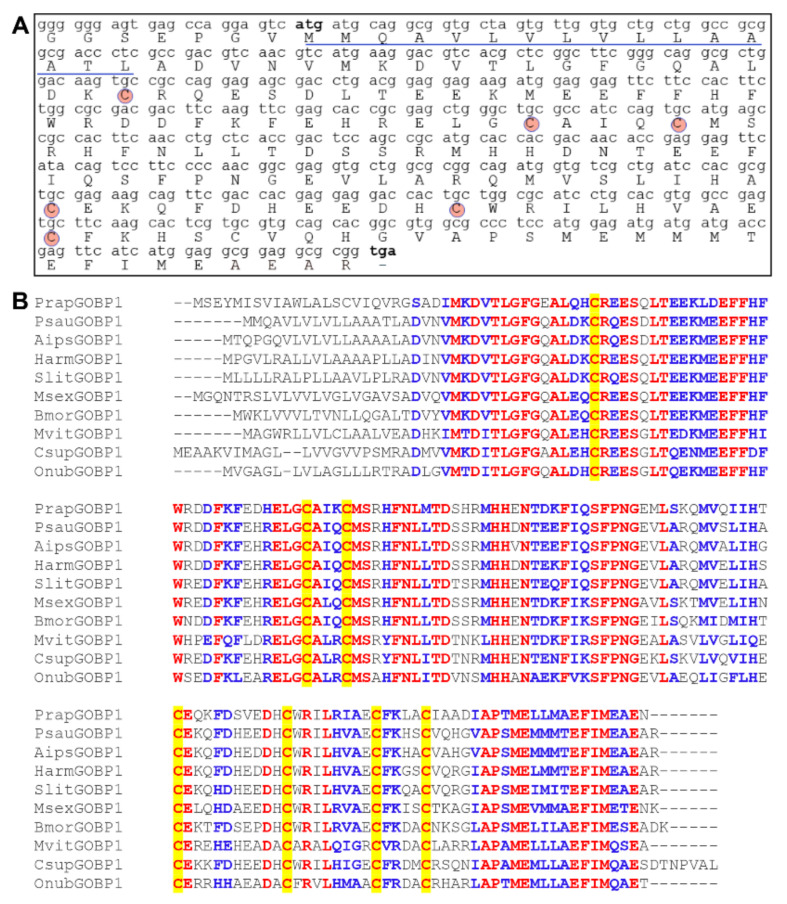
PsauGOBP1 sequence characterization and multiple alignment with other homologous proteins. (**A**) Nucleotide sequence and deduced amino acid sequence of the PsauGOBP1 cDNA. The six conserved cysteine residues are indicated in circles. The predicted signal peptide is underlined. The start and stop codons are marked in bold. (**B**) Alignment of GOBP1s of 10 Lepidoptera species. *Pieris rapae* (PrapGOBP1, XP_022118428.1); *Peridroma saucia* (PsauGOBP1, MW013058.1); *Agrotis ipsilon* (AipsGOBP1, AFM36759.1); *Helicoverpa armigera* (HarmGOBP1, XP_021192665); *Spodoptera litura* (SlitGOBP1, XP_022816701.1); *Manduca sexta* (MsexGOBP1, XP_030028623.1); *Bombyx mori* (BmorGOBP1, CAA64444); *Maruca vitrata* (MvitGOBP1, ALM04194); *Chilo suppressalis* (CsupGOBP1, ACJ07126); *Ostrinia nubilalis* (OnubGOBP1, BBB15959.1). The six highly conserved cysteine residues in the GOBP1s are shaded in yellow. Strictly identical residues are highlighted with red letters. Residues with similar physicochemical properties are shown in blue letters.

The full-length cDNA of *PsauGOBP1* (GenBank number: MW013058.1) was cloned by RT-PCR using gene-specific primers. The open reading frame (ORF) of *PsauGOBP1* has 489 base pairs and encodes a predicted precursor protein of 162 amino acids. The initial 16 amino acid residues were predicted as the signal peptide by SignalP-5.0 software (Figure 1A). The calculated molecular weight of the mature PsauGOBP1 is 17.2 kDa, and its isoelectric point is 5.09. An alignment of the amino acid sequences of PsauGOBP1 with orthologous proteins from nine other insects revealed that GOBP1 is highly conserved among lepidopterans. GOBP1s from the 10 assessed Lepidoptera species (including PsauGOBP1) have the typical six-cysteine signature and fit the following motif pattern of the OBP family: C_1_-X_15-39_-C_2_-X_3_-C_3_-X_21-44_-C_4_-X_7-15_-C_5_-X_8_-C_6_ (Figure 1B) [72,73]. PsauGOBP1 shares high identity (58–95%) with other lepidopteran GOBP1s. The highest identity is 95% with *A. ipsilon*.

### 3.2. Phylogenetic Analyses

**Figure 2:**
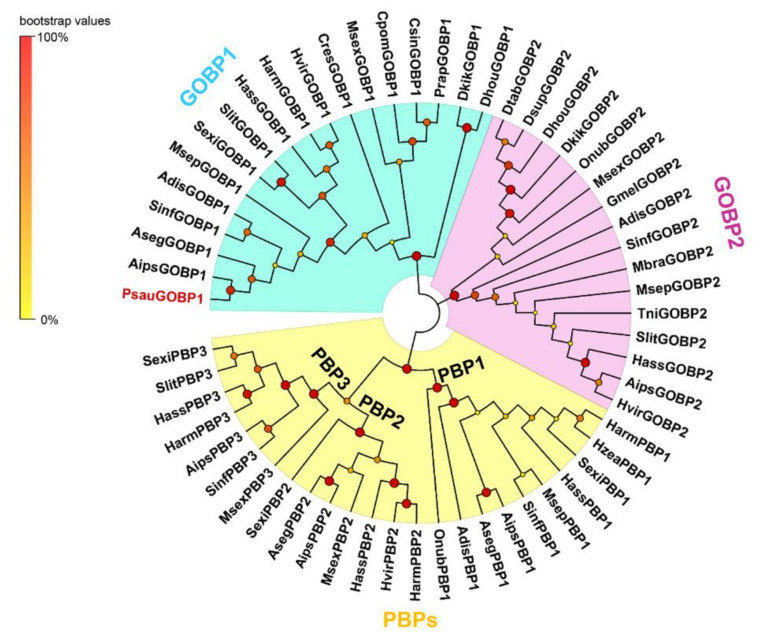
Phylogenetic tree of PsauGOBP1 with GOBPs and PBPs from other Lepidoptera species. The unrooted neighbor-joining tree was constructed by the MEGA7.0 program with the Jones–Taylor–Thornton (JTT) matrix-based method. Node support was estimated with 1000 bootstrap replicates, and the bootstrap values are indicated by the size and color of circles at the branch nodes based on the scale at the top left. The protein accession numbers of all GOBPs and PBPs used in the phylogenetic tree construction are provided in Table S2.

We selected 34 GOBPs and 24 PBPs from 25 Lepidoptera species and analyzed their phylogenetic relationships in a neighbor-joining tree. The results revealed that Lepidoptera GOBP groups clearly separated from Lepidoptera PBP groups. Furthermore, GOBP1 and GOBP2 subfamilies are well separated from each other, and the PBP clade is divided into three distinct groups (PBP1, PBP2, and PBP3). The phylogenetic analysis also showed that PsauGOBP1 is closely clustered with their orthologs from *A. ipsilon* and *A. segetum* (Figure 2).

### 3.3. Expression Patterns of PsauGOBP1 in Different Tissues of Peridroma saucia

**Figure 3:**
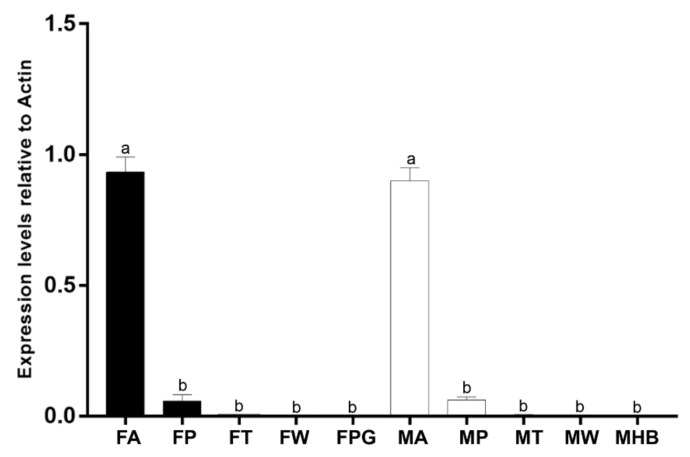
Expression patterns of *PsauGOBP1* in different tissues of *P*. *saucia*. RT-qPCR analyses were conducted for *PsauGOBP1* in the following tissues: female antennae (FA); female proboscises (-FP); female tarsi (FT); female wings (FW); female pheromone glands (FPG); male antennae (MA); male proboscises (MP); male tarsi (MT); male wings (MW); male hair brushes (MHB). Values are means + SE (n = 3). Means with different letters are significantly different (*p* < 0.05) according to a one-way ANOVA followed by a Tukey multiple comparison test.

The expression profiles of PsauGOBP1 in different *P*. *saucia* tissues were investigated using RT-qPCR. By comparing the expression levels in antennae, proboscises, tarsi, wings, pheromone glands, and hair brushes of both sexes, we found that PsauGOBP1was significantly expressed in the antennae of both males and females. Interestingly, the expression of PsauGOBP1 was also detected in the taste organ, i.e., the proboscis, of both sexes (Figure 3).

### 3.4. Bacterial Expression and Purification of Recombinant PsauGOBP1

**Figure 4:**
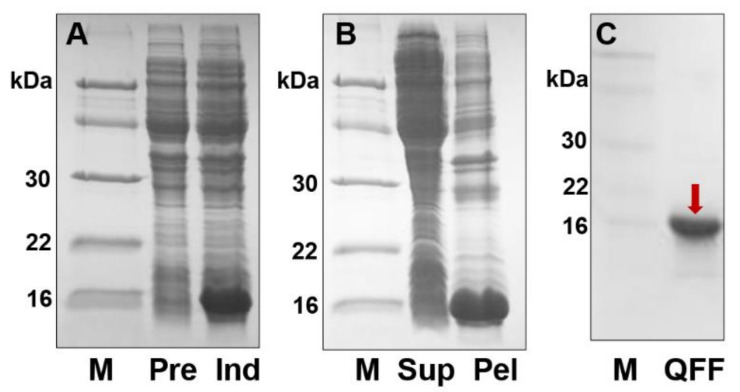
Expression and purification of the recombinant PsauGOBP1. (**A**) SDS-PAGE analysis relative to crude bacterial extracts before (Pre) and after (Ind) induction with IPTG; (**B**) the supernatant (Sup) and the bacterial pellet (Pel) after sonication and centrifugation; (**C**) purification of recombinant PsauGOBP1 by anion-exchange chromatography on QFF. The targeted protein is indicated by a red arrow. Molecular weight markers (M) are, from the top, 66, 45, 30, 22, and 16 kDa.

To investigate PsauGOBP1 at the protein level, PsauGOBP1 was expressed in a bacterial system using the vector pET30b, which did not contain any modifications with respect to the mature sequence apart from the addition of an initial methionine. The recombinant PsauGOBP1 was produced in high yields (about 25 mg/L) as insoluble inclusion bodies. Solubilization was accomplished by denaturation and refolding according to previously reported protocols [74]. Purification was performed by anion-exchange chromatography on QFF columns, and an expected target band of ~17 kDa was finally obtained (Figure 4).

### 3.5. Western Blot Analysis of PsauGOBP1 in P. saucia Antennae

**Figure 5:**
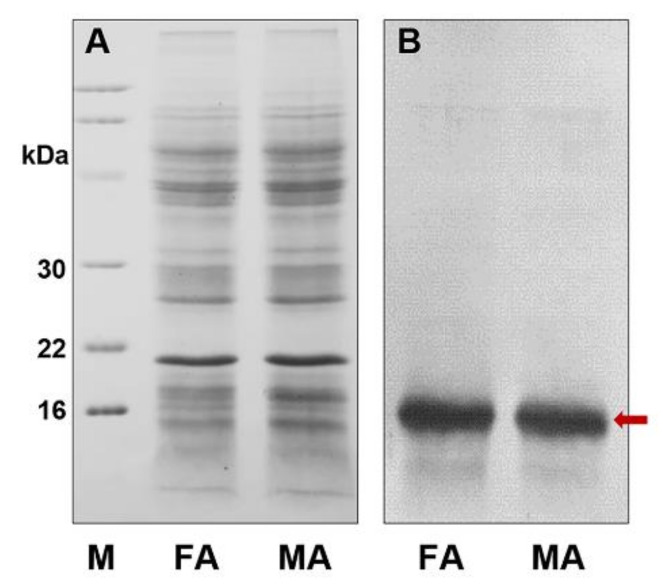
SDS-PAGE and Western blot of extracts from male and female antennae of *Peridroma saucia* adults. (**A**) SDS-PAGE; (**B**) Western blot. FA: female antennae; MA: male antennae. Expression of PsauGOBP1 does not significantly differ in male antennae vs. female antennae. Target proteins are indicated by a red arrow. Molecular weight markers (M) are, from the top, 94, 66, 45, 30, 22, and 16 kDa.

This expression pattern of PsauGOBP1 in the antennae was validated at the protein level by Western blot analysis. Using extracts from antennae, we detected the protein with no significant difference between males and females (Figure 5).

### 3.6. Fluorescence Binding Assay

**Figure 6:**
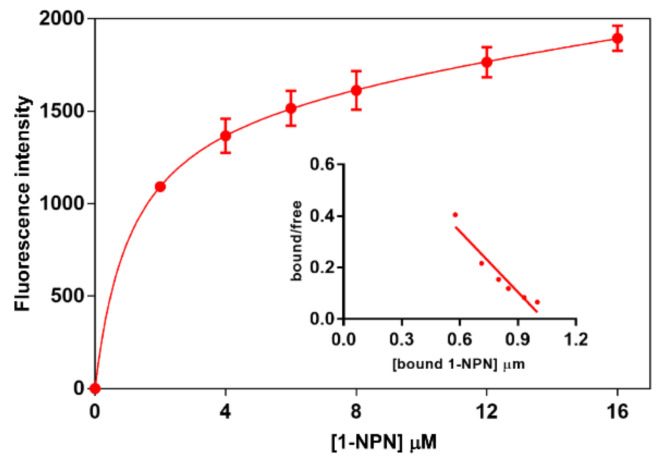
Affinity of 1-NPN to the recombinant protein PsauGOBP1. A 2 mM solution of the protein in Tris was titrated with a 1 mM solution of 1-NPN in methanol to final concentrations of 2–16 µM. Analysis of the means of three replicates by Prism software indicated the presence of a single binding site with a dissociation constant of 1.9 µM.

**Figure 7:**
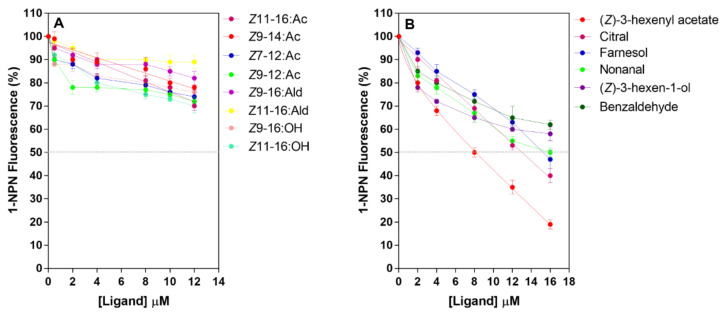
Competitive-binding assays of selected ligands to the recombinant protein PsauGOBP1. (**A**) Moth sex pheromones; (**B**) selected host plant volatiles. In each assay, a mixture of the protein and 1-NPN in Tris, both at 2 mM, was titrated with the competing ligand to final concentrations of 12 µM (sex pheromone components) or 16 µM (host plant volatiles). Affinities of 8 sex pheromone components and 26 host plant volatiles were tested, and the data for all of the tested ligands are reported in Table 1.

To assess the binding ability of PsauGOBP1, we first measured its affinity to the fluorescent probe 1-NPN. The results showed that 1-NPN bound PsauGOBP1 with a dissociation constant of 1.9 µM (Figure 6). Affinities of other ligands were then evaluated in competitive-binding experiments. We tested 34 synthetic compounds as competitors, including 2 sex pheromone components of *P*. *saucia*, 6 sex pheromone components of other moths, and 26 host plant volatiles. The results revealed that PsauGOBP1 had the highest affinity to (*Z*)-3-hexenyl acetate, with a K_D_ value of 4.0 ± 0.1 μM. Three other host plant volatiles, i.e., citral, farnesol, and nonanal, had moderate binding affinities, with K_D_ values of 5.6 ± 0.4 μM, 6.4 ± 0.6 μM, and 6.8 ± 0.3 μM, respectively. Binding affinities were weak for (*Z*)-3-hexen-1-ol and benzaldehyde, with the K_D_ values of 8.5 ± 0.6 and 9.4 ± 0.5 μM, respectively (Figure 7, Table 1). Other chemicals tested in the experiment did not bind to PsauGOBP1 (K_D_ > 20 μM). The binding affinities of all tested ligands are listed in Table 1.

### 3.7. Electroantennogram (EAG) Recording

**Figure 8:**
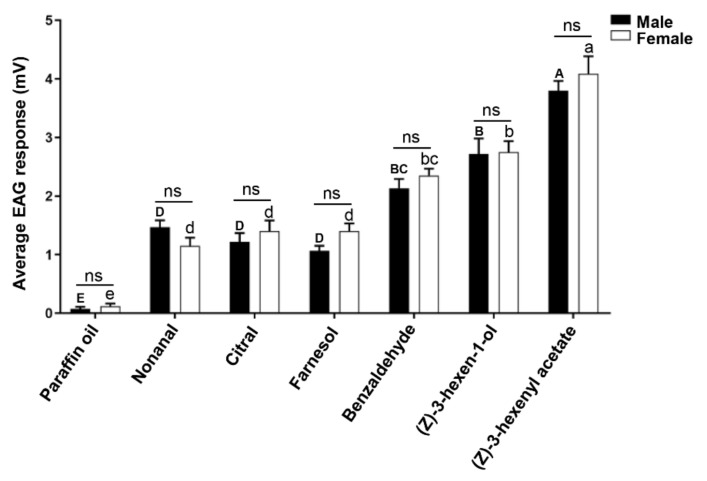
EAG responses of male and female *Peridroma saucia* to host plant volatiles. Two- to three-day-old males and females of *P*. *saucia* were used for EAG tests with six host plant volatiles that had substantial binding affinities to PsauGOBP1 in the competitive-binding assays. The dose of each tested compound was 100 µg, and unpaired Student’s *t*-test indicated that the EAG responses did not significantly differ (*p* > 0.05) between male and female antennae (as indicated by “ns”). Values above bars are means + SE (n = 20). According to one-way ANOVA followed by the Tukey multiple comparison test, means with different uppercase letters indicate significantly different (*p* < 0.05) responses of male antennae, and means with different lowercase letters indicate significantly different responses (*p* < 0.05) of female antennae.

We selected the six host plant volatiles that bound to PsauGOBP1 in the competitive-binding assays for EAG analysis. The results demonstrated that all six of the tested compounds elicited electrophysiological responses from the *P*. *saucia* antennae when compared with the control group (paraffin oil), and the responses were not statistically different between males and females. (*Z*)-3-hexenyl acetate, which had the highest affinity with PsauGOBP1 in competitive-binding assays, elicited the strongest EAG responses from both male and female antennae. (*Z*)-3-hexen-1-ol and benzaldehyde also elicited strong responses from *P*. *saucia* antennae in spite of its weak affinity to PsauGOBP1. In contrast, three ligands with moderate affinities, i.e., citral, farnesol, and nonanal, had weak EAG responses (Figure 8).

### 3.8. Behavioral Trials

**Figure 9:**
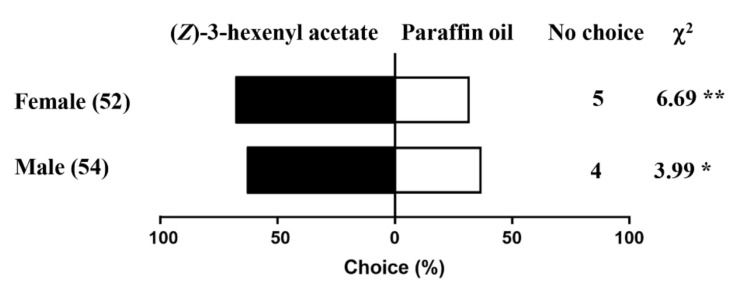
Behavioral responses of male and female *Peridroma saucia* to (*Z*)-3-hexenyl acetate in a Y-tube olfactometer. If a parasitoid did not make a choice, it was removed and recorded as “no choice”. Numbers in brackets represent sample sizes (*chi*-squared test, ** *p* < 0.01; * *p* < 0.05). Moths that made “no choice” were not included in the statistical analysis.

As (*Z*)-3-hexenyl acetate had the highest affinity with recombinant PsauGOBP1 and also elicited the strongest EAG responses in *P*. *saucia* antennae, we assessed the behavioral responses of *P*. *saucia* to this volatile in a Y-tube olfactometer. The results showed that both males and females of *P*. *saucia* were attracted to (*Z*)-3-hexenyl acetate (χ^2^_male_ = 3.99, *p* = 0.045; χ^2^_female_ = 6.69, *p* = 0.009). The percentage of males and females of *P*. *saucia* showing chemotaxis to (*Z*)-3-hexenyl acetate was 64% and 68%, respectively (Figure 9).

## 4. Discussion

Odorant-binding proteins are important mediators of insect chemoreception [75,76,77]. In this study, RT-PCR was used to clone the full-length of *GOBP1* cDNA from the antennae of *P*. *saucia*. Amino acid sequence characterization coupled with phylogenetic analyses verified that this protein should be classified in the Lepidoptera GOBP1 subfamily [34].

Expression profile analyses using RT-qPCR revealed that PsauGOBP1 was expressed specifically at a very high level in female and male antennae, which indicates that PsauGOBP1 is probably involved in chemoreception. The expression levels were significantly lower in the proboscises, tarsi, wings, pheromone glands, and hair brushes of *P*. *saucia* than in antennae, which is consistent with the expression patterns of GOBPs in other Lepidoptera species [78,79,80]. RT-qPCR combined with Western blot analysis demonstrated that the PsauGOBP1 expression level was equivalent in male vs. female antennae. Similarly, the GOBP1 of *Spodoptera litura* was expressed at equal levels in the antennae of both sexes [42]. The GOBP1 of *Sesamia nonagrioides*, however, was expressed at higher levels in females than in males, although expression levels were also high in males [81]. In contrast, the transcript level of GOBP1 in *Grapholita molesta* was higher in male antennae than in female antennae [82]. These different findings may be due to the plasticity of the insect physiological state or to the differentiation of the protein function during the long evolutionary history of these species.

As carriers of odorant molecules that activate the membrane-bound olfactory receptors (ORs), the ligand-binding specificity of OBPs may substantially contribute to the specificity of the sensilla [83]. To determine the binding specificities of PsauGOBP1, we assessed 34 synthetic volatiles as ligands in binding assays. These 34 included the volatiles emitted by maize, cotton, tobacco, and soybean [71,84,85,86,87]; female sex pheromone components of *P*. *saucia* [51,52]; female sex pheromone components of other Noctuidae species including *H*. *armigera*, *Mythimna separata*, *Spodoptera frugiperda*, and *A*. *ipsilon* [88,89,90,91,92]. Among the tested ligands, (*Z*)-3-hexenyl acetate had the highest binding affinity (K_D_ = 4.0 ± 0.1 μM) to PsauGOBP1. PsauGOBP1 also had binding affinities with five plant volatiles including citral (K_D_ = 5.6 ± 0.4 μM), farnesol (K_D_ = 6.4 ± 0.6 μM), nonanal (K_D_ = 6.8 ± 0.3 μM), (*Z*)-3-hexen-1-ol (K_D_ = 8.5 ± 0.6 μM), and benzaldehyde (K_D_ = 9.4 ± 0.5 μM). Although previous studies with *S. exigua*, *A*. *ipsilon*, and *Plutella xylostella* showed that GOBP1 could bind to sex pheromone components [40,44,47], PsauGOBP1 did not show binding affinities for female sex pheromone components of its conspecific species or other closely related moth species (K_D_ > 20 μM). Similar findings were reported for *Maruca vitrata*, *Loxostege sticticalis*, and *Athetis lepigone*, whose GOBP1s only bound to plant volatiles [43,48,93]. Such divergence indicates that the function of GOBP1s may differ among species. Further in vivo functional analyses, including gene knockdown studies, are needed to determine the functions of the GOBP1s.

We measured the EAG responses of adult *P*. *saucia* to six plant volatiles that were found to bind with recombinant PsauGOBP1 in our competitive-binding assays. The results showed that the electrophysiological responses of antennae did not differ between males and females of *P*. *saucia* but did differ among the volatiles. The strongest EAG response of both male and female antennae was to (*Z*)-3-hexenyl acetate, which also had the highest binding affinity to PsauGOBP1 among the tested compounds. This suggests that PsauGOBP1 may act as the major carrier of (*Z*)-3-hexenyl acetate in *P*. *saucia* antennae. EAG responses were weak for the three volatiles with moderate binding affinities (citral, nonanal, and farnesol) but were relatively strong for the two compounds with low binding affinities, i.e., (*Z*)-3-hexen-1-ol and benzaldehyde. These results are consistent with recent findings that CsupGOBP1 of *Chilo suppressalis* has strong binding affinities to volatiles that elicited weak antennal responses [45]. It is not surprising, however, that the results of the fluorescence competitive binding assay do not always correlate well with the physiological activity of the ligands tested, because one odorant may bind to multiple OBPs.

As (*Z*)-3-hexenyl acetate was the strongest ligand of recombinant PsauGOBP1 and the strongest EAG stimulator among the tested compounds, we investigated its effects on *P*. *saucia* behavior. In choice tests between (*Z*)-3-hexenyl acetate and paraffin oil (odorant solvent) in a Y-tube olfactometer, both male and female *P*. *saucia* showed a significant attraction to (*Z*)-3-hexenyl acetate. As a green leaf volatile, (*Z*)-3-hexenyl acetate is produced by a wide range of plant species (e.g., maize, cotton, tobacco, and soybean) [94,95,96,97]. It has been reported that (*Z*)-3-hexenyl acetate could prime inducible production of sesquiterpenes and jasmonic acid in undamaged corn seedlings [98]. Sun et al. reported that (*Z*)-3-hexenyl acetate attracted *H*. *armigera* in wind tunnels [99]. Piesik et al. reported that *Cephus cinctus* females are attracted to some concentrations of (*Z*)-3-hexenyl acetate [100]. It also significantly enhanced the capture of male *H. zea* and *Cydia pomonella* in field experiments, when mixed with sex pheromone components [101]. In another experiment, the mixture of (Z)-3-hexenyl acetate and the sex pheromone components in a 1:1 ratio, markedly increased the captures of male and female *P*. *xylostella* in trap assays [102]. (*Z*)-3-hexenyl acetate is one of the six green-leaf odors that play a main role in attracting the mated female *S*. *littoralis* to the cotton [103]. Results of the current study suggest that both males and females of *P*. *saucia* may use (*Z*)-3-hexenyl acetate to locate appropriate mating or feeding sites, and that PsauGOBP1 may mediate the interaction between the chemosensory system of *P*. *saucia* and (*Z*)-3-hexenyl acetate. Based on these results, we suggest two directions for future studies: (1) RNAi experiments to validate the role of PsauGOBP1 in the detection of (*Z*)-3-hexenyl acetate and to determine how (*Z*)-3-hexenyl acetate influences *P*. *saucia* behavior; (2) experiments that explore how (*Z*)-3-hexenyl acetate could be used as lures for monitoring or other odor-based strategies in *P*. *saucia* control.

## 5. Conclusions

In this study, we found that *P*. *saucia* GOBP1 binds to host-plant volatiles and that binding was strongest to (*Z*)-3-hexenyl acetate. EAG tests demonstrated that (*Z*)-3-hexenyl acetate elicits strong responses from the antennae of both males and females of *P*. *saucia*. Y-tube olfactometer bioassays showed that (*Z*)-3-hexenyl acetate is attractive to both males and females of *P*. *saucia*. These results increase our understanding of the olfactory mechanism of *P*. *saucia* and provides a theoretical basis for the design of attractants for the control of *P*. *saucia*.

## Figures and Tables

**Table 1:** Binding capability of recombinant PsauGOBP1 to tested odorants.

Ligands	Maximum Concentration	Fluorescence (%) at Maximum Concentration	IC_50_ * (μM)	K_D_ * (μM)
*P*. *saucia* sex pheromones				
*Z*11-16: Ac	12	70 ± 2	>20	-
*Z*9-14: Ac	12	78 ± 2	>20	-
Other moth sex pheromones				
*Z*11-16: Ald	12	89 ± 1	>20	-
*Z*9-16: Ald	12	82 ± 3	>20	-
*Z*7-12: Ac	12	74 ± 4	>20	-
*Z*9-12: Ac	12	72 ± 2	>20	-
*Z*11-16: OH	12	70 ± 5	>20	-
*Z*9-16: OH	12	76 ± 1	>20	-
Host plant volatiles				
(*Z*)-3-hexenyl acetate	16	19 ± 2	8.2 ± 0.2	4.0 ± 0.1
(*E*)-2-hexenyl acetate	16	77 ± 7	>20	-
Methyl jasmonate	16	80 ± 5	>20	-
Methyl salicylate	16	73 ± 3	>20	-
Phenylethyl acetate	16	64 ± 2	>20	-
Octanal	16	61 ± 6	>20	-
Decanal	16	85 ± 2	>20	-
Nonanal	16	50 ± 2	13.9 ± 0.6	6.8 ± 0.3
Citral	16	40 ± 3	11.5 ± 1.0	5.6 ± 0.4
(*E*)-2-hexenal	16	64 ± 4	>20	-
Benzaldehyde	16	62 ± 2	19.3 ± 1.1	9.4 ± 0.5
Heptanol	16	65 ± 4	>20	-
Farnesol	16	47 ± 4	13.1 ± 1.2	6.4 ± 0.6
(*Z*)-3-hexen-1-ol	16	58 ± 3	17.4 ± 1.5	8.5 ± 0.6
(*E*)-2-hexen-1-ol	16	67 ± 2	>20	-
Dodecanol	16	75 ± 8	>20	-
Linalool	16	79 ± 2	>20	-
*β*-myrcene	16	94 ± 1	>20	-
*β*-pinene	16	100 ± 2	>20	-
*D*-limonene	16	100 ± 5	>20	-
(*E*)-β-farnesene	16	87 ± 4	>20	-
Ocimene	16	74 ± 7	>20	-
(*E*)-caryophyllene	16	75 ± 1	>20	-
Jasmonic acid	16	68 ± 3	>20	-
(*Z*)-jasmone	16	83 ± 5	>20	-
Indole	16	70 ± 9	>20	-

We consider PsauGOBP1 had no binding with the tested ligands if the IC50 values > 20 μM and K_D_ values were not to be calculated and are represented as “-”. Data are means of three independent experiments and represents mean ± SE. IC_50_ *: the concentration of ligands halving the initial fluorescence value; K_D_ *: the calculated dissociation constants.

## Data Availability

The authors confirm that the data supporting the findings of this study are available within the article and its Supplementary Materials.

## Supplementary Materials

The following are available online at https://www.mdpi.com/article/10.3390/insects12100939/s1, Table S1. Nucleotide primers used in this article. Table S2. The protein accession numbers of GOBP and PBP sequences used in the phylogenetic tree construction. Table S3. Odorants for fluorescence binding assays of recombinant PsauGOBP1.
