# Supplementary material for: GOBP1 from the Variegated Cutworm Peridroma saucia (Hübner) (Lepidoptera: Noctuidae) Displays High Binding Affinities to the Behavioral Attractant (Z)-3-Hexenyl acetate
Source: Insects. 2021 Oct 15;12(10):939. doi: 10.3390/insects12100939 (PMC8540349; doi:10.3390/insects12100939)
Supplement: Supplementary file 1 [file insects-12-00939-s001.zip › Supplementary table 1.pdf]

**Table S1**. Nucleotide primers used in this article.

| Primer name | Sequence (5′-3′) |
|---|---|
| **Cloning CDS** | |
| PsauGOBP1-F | ATGATGCAGGCGGTGCTAGTG |
| PsauGOBP1-R | TCACCGCGCCTCCGCCTCCAT |
| **RT-qPCR** | |
| PsauGOBP1-F | CCACTTCAACCTGCTCACC |
| PsauGOBP1-R | AACTCGGTCATCTACATCTC |
| PsauActin-F | TCATCACCATCGGAAACGAAC |
| PsauActin-R | GCGTACAAGTCCTTACGGAT |
| **Recombinant PsauGOBP1 expression** | |
| PsauGOBP1-F | CC*CATATG*GCCGACGTCAACGTCATGAAG |
| PsauGOBP1-R | AT*GAATTC*TCACCGCGCCTCCGCCT |

F: forward primer; R: reverse primer; CDS: coding sequence; PsauActin: β-actin protein of *P. saucia.* Underlined showed the NdeI (CATATG) and EcoRI (GAATTC) restriction enzyme sites in the forward and reverse primer, respectively.
