# Supplementary material for: GOBP1 from the Variegated Cutworm Peridroma saucia (Hübner) (Lepidoptera: Noctuidae) Displays High Binding Affinities to the Behavioral Attractant (Z)-3-Hexenyl acetate
Source: Insects. 2021 Oct 15;12(10):939. doi: 10.3390/insects12100939 (PMC8540349; doi:10.3390/insects12100939)
Supplement: Supplementary file 1 [file insects-12-00939-s001.zip › Supplementary table 2.pdf]

Table S2. The protein accession numbers of GOBP and PBP sequences used in the phylogenetic tree construction.

Protein	Accession number	Species
PsauGOBP1	MW013058.1	*Peridroma Saucia*
MsepGOBP1	AWT22240.1	*Mythimna separata*
AipsGOBP1	AFM36759.1	*Agrotis ipsilon*
AsegGOBP1	ABI24159.1	*Agrotis segetum*
HarmGOBP1	XP_021192665.1	*Helicoverpa armigera*
HvirGOBP1	PCG66043.1	*Heliothis viresence*
SexiGOBP1	ACY78412.1	*Spodoptera exigua*
SinfGOBP1	AGS36742.1	*Sesamia inferens*
SlitGOBP1	XP_022816701.1	*Spodoptera litura*
HassGOBP1	AAW65076.1	*Helicoverpa assulta*
AdisGOBP1	ALJ93806.1	*Athetis dissimilis*
MsexGOBP1	XP_030028623.1	*Manduca sexta*
CpomGOBP1	AFP66957.1	*Cydia pomonella*
CsinGOBP1	AHY86493.1	*Conopomorpha sinensis*
CresGOBP1	AXN76737.1	*Clostera restitura*
PrapGOBP1	XP_022118428.1	*Pieris rapae*
DkikGOBP1	AGJ83357.1	*Dendrolimus kikuchii*
DhouGOBP1	AGJ83358.1	*Dendrolimus houi*
AipsGOBP2	AAP57462.1	*Agrotis ipsilon*
DsupGOBP2	AGJ83351.1	*Dendrolimus superans*
HassGOBP2	AAQ54909.1	*Helicoverpa assulta*
HvirGOBP2	PCG76987.1	*Heliothis viresence*
SlitGOBP2	XP_022817877.1	*Spodoptera litura*
MsepGOBP2	AWT22242.1	*Mythimna separata*
AdisGOBP2	ALJ93807.1	*Athetis dissimilis*
DkikGOBP2	AGJ83353.1	*Dendrolimus kikuchii*
MbraGOBP2	AAC05703.2	*Mamestra brassicae*
TniGOBP2	XP_026739044.1	*Trichoplusia ni*
SnifGOBP2	AHC72380.1	*Sesamia inferens*
MsexGOBP2	XP_030025611.1	*Manduca sexta*
DtabGOBP2	AGJ71277.1	*Dendrolimus tabulaeformis*
OnubGOBP2	BBB15978.1	*Ostrinia nubilalis*
GmelGOBP2	QEI46781.1	*Galleria mellonella*
DhouGOBP2	AGJ83354.1	*Dendrolimus houi*
HarmPBP1	HQ436362.1	*Helicoverpa armigera*
HzeaPBP1	AF090191.1	*Helicoverpa zea*
SexiPBP1	AAS55551.2	*Spodoptera exigua*
HassPBP1	AAW65077.1	*Helicoverpa assulta*
MsepPBP1	BAG71416.1	*Mythimna separata*

SinfPBP1	AEX58642.1	*Sesamia inferens*
AipsPBP1	AFM36756.1	*Agrotis ipsilon*
AsegPBP1	AAD41276.1	*Agrotis segetum*
AdisPBP1	ALJ93809.1	*Athetis dissimilis*
OnubPBP1	AAD39447.1	*Ostrinia nubilalis*
HarmPBP2	AEB54583.1	*Helicoverpa armigera*
HvirPBP2	CAL48346.1	*Heliothis virescens*
HassPBP2	ABY28381.2	*Helicoverpa assulta*
AipsPBP2	AFM36757.1	*Agrotis ipsilon*
MsexPBP2	AAF16710.1	*Manduca sexta*
AsegPBP2	AAX85460.1	*Agrotis segetum*
SexiPBP2	ABK41046.1	*Spodoptera exigua*
SexiPBP3	ACY78413.1	*Spodoptera exigua*
SlitPBP3	AIS72934.1	*Spodoptera litura*
HassPBP3	ABB91374.1	*Helicoverpa assulta*
HarmPBP3	AAO16091.1	*Helicoverpa armigera*
AipsPBP3	AFM36758.1	*Agrotis ipsilon*
SinfPBP3	AEQ30020.1	*Sesamia inferens*
MsexPBP3	AAF16703.1	*Manduca sexta*
