# Supplementary material for: GOBP1 from the Variegated Cutworm Peridroma saucia (Hübner) (Lepidoptera: Noctuidae) Displays High Binding Affinities to the Behavioral Attractant (Z)-3-Hexenyl acetate
Source: Insects. 2021 Oct 15;12(10):939. doi: 10.3390/insects12100939 (PMC8540349; doi:10.3390/insects12100939)
Supplement: Supplementary file 1 [file insects-12-00939-s001.zip › Supplementary table 3.pdf]

Table S3. Odorants for fluorescence binding assays of recombinant PsauGOBP1.

Odorant	CAS number*	Purity (%)	Company
***P. saucia* sex pheromones**			
*Z*11-16: Ac	34010-21-4	≥91	J&K Scientific
*Z*9-14: Ac	16725-53-4	≥93	J&K Scientific
**Other moth sex pheromones**			
*Z*11-16: Ald	53939-28-9	≥95	TCI
*Z*9-16: Ald	56219-04-6	≥95	Fluorochem
*Z*7-12: Ac	14959-86-5	≥91	J&K Scientific
*Z*9-12: Ac	16974-11-1	≥93	J&K Scientific
*Z*11-16: OH	56683-54-6	≥91	J&K Scientific
*Z*9-16: OH	10378-01-5	≥98	Sigma-Aldrich
**Host plant volatiles**			
(*Z*)-3-hexenyl acetate	3681-71-8	≥99	J&K Scientific
(*E*)-2-hexenyl acetate	2497-18-9	≥97	TCI
Methyl jasmonate	39924-52-2	≥95	Fluorochem
Methyl salicylate	119-36-8	≥99	J&K Scientific
Phenylethyl acetate	103-45-7	≥98	TCI
Octanal	124-13-0	≥98	TCI
Decanal	112-31-2	≥97	TCI
Nonanal	124-19-6	≥95	TCI
Citral	5392-40-5	≥96	TCI
(*E*)-2-hexenal	6728-26-3	≥97	J&K Scientific
Benzaldehyde	100-52-7	≥98	J&K Scientific
Heptanol	111-70-6	≥98	TCI
Farnesol	4602-84-0	≥96	J&K Scientific
(*Z*)-3-hexen-1-ol	928-96-1	≥98	J&K Scientific
(*E*)-2-hexen-1-ol	928-95-0	≥96	J&K Scientific
Dodecanol	112-53-8	≥99	TCI
Linalool	78-70-6	≥98	J&K Scientific
β-myrcene	123-35-3	≥90	TRC
β-pinene	127-91-3	≥95	J&K Scientific
D-Limonene	5989-27-5	≥95	Fluorochem
(*E*)-β-farnesene	18794-84-8	≥95	Macklin
β-ocimene	13877-91-3	≥90	TRC
(*E*)-caryophyllene	87-44-5	≥90	TCI
Jasmonic acid	77026-92-7	≥90	Sigma-Aldrich
(*Z*)-jasmone	488-10-8	≥93.5	J&K Scientific
Indole	120-72-9	≥99	J&K Scientific

*CAS number: chemical abstracts service number.
